# Surgical Efficacy and Safety of Patients with Locally Advanced Gastric Cancer following Neoadjuvant Concurrent Chemoradiotherapy and Chemotherapy

**DOI:** 10.1155/2022/3719241

**Published:** 2022-03-19

**Authors:** Ching-Chun Li, Yung-Sung Yeh, Yen-Cheng Chen, Wei-Chih Su, Tsung-Kun Chang, Hsiang-Lin Tsai, Ching-Wen Huang, Cheng-Jen Ma, Tzu-Chieh Yin, Po-Jung Chen, Jaw-Yuan Wang

**Affiliations:** ^1^Division of Colorectal Surgery, Department of Surgery, Kaohsiung Medical University Hospital, Kaohsiung Medical University, Kaohsiung, Taiwan; ^2^Division of Trauma and Surgical Critical Care, Department of Surgery, Kaohsiung Medical University Hospital, Kaohsiung Medical University, Kaohsiung, Taiwan; ^3^Graduate Institute of Clinical Medicine, College of Medicine, Kaohsiung Medical University, Surgery, Taiwan; ^4^Department of Surgery, Faculty of Medicine, College of Medicine, Kaohsiung Medical University, Kaohsiung, Taiwan; ^5^Division of General and Digestive Surgery, Department of Surgery, Kaohsiung Medical University Hospital, Kaohsiung Medical University, Kaohsiung, Taiwan; ^6^Department of Surgery, Kaohsiung Municipal Tatung Hospital, Kaohsiung Medical University, Kaohsiung, Taiwan; ^7^Graduate Institute of Medicine, College of Medicine, Kaohsiung Medical University, Kaohsiung, Taiwan; ^8^Center for Cancer Research, Kaohsiung Medical University, Kaohsiung, Taiwan; ^9^Center for Liquid Biopsy and Cohort Research, Kaohsiung Medical University, Kaohsiung, Taiwan; ^10^Pingtung Hospital, Ministry of Health and Welfare, Pingtung, Taiwan

## Abstract

**Background:**

The safety and efficacy of gastrectomy for locally advanced gastric cancer (LAGC) following neoadjuvant therapy have gained increasing attention. In this article, we present our preliminary treatment results and compare the surgical safety and outcomes of neoadjuvant concurrent chemoradiotherapy (CCRT) with those of chemotherapy in patients with LAGC. *Patients and Methods*. Sixty-three patients with a diagnosis of LAGC (clinical staging cT3N2+, cT4aN+, or cT4b) who had received neoadjuvant therapy at any period from January 2014 to December 2020 were enrolled. Among 63 patients who received neoadjuvant therapy, 38 were treated with CCRT and 25 were treated with chemotherapy. They regularly received follow-up until July 2021. The patients' characteristics, including their clinical data, perioperative results, and pathologic outcomes, were analyzed.

**Results:**

The CCRT and chemotherapy groups did not significantly differ with respect to age, sex, or clinical stage (all *p* > 0.05). Finally, radical gastrectomy was performed in 15 (39.5%) patients with neoadjuvant CCRT and 10 (40.0%) patients with neoadjuvant chemotherapy. Both groups did not significantly differ with respect to operation time, blood loss, operative morbidities, or postoperative length of stay (both *p* > 0.05). The patients in the CCRT group exhibited favorable pathologic responses after treatment: three patients exhibited a pathologic complete response (pCR) and four, seven, and one patients exhibited a response at pathologic stages I, II, and III, respectively. By contrast, among the patients in the chemotherapy group after treatment, one patient exhibited a pCR and one, four, and four patients exhibited a response at pathologic stages I, II, and III, respectively.

**Conclusions:**

Radical resection in patients with LAGC is challenging. This study reports that neoadjuvant CCRT is associated with better pathologic response with no increase in serious postoperative complications. However, further prospective randomized trials involving patients with LAGC receiving neoadjuvant CCRT should be conducted to verify the findings of this retrospective study.

## 1. Introduction

Gastric cancer is a major cause of cancer-related deaths worldwide; over 1 million new cases are diagnosed annually [1]. Although survival improves if gastric cancer is detected early, the mortality and morbidity rates of locally advanced or distant metastatic gastric cancer, occurring in up to 60% of patients, remain high [2, 3]. In patients with unresectable or borderline resectable locally advanced gastric cancer (LAGC), complete surgical resection is the only technique with the prospect of curing the disease; moreover, multimodal treatment is implemented to improve the patient's survival chances [4]. According to the MAGIC (Medical Research Council Adjuvant Gastric Infusional Chemotherapy) study, neoadjuvant chemotherapy has been the treatment of choice for gastric cancer [5]. The advantages of neoadjuvant chemotherapy and perioperative chemotherapy include tumor downstaging and improved overall survival [5, 6].

Several studies have demonstrated that postoperative chemoradiotherapy can improve survival in patients with advanced gastric or gastroesophageal junction cancer [4, 5]. In our previous retrospective study involving patients with unresectable and locally advanced or metastatic gastric cancer, neoadjuvant concurrent chemoradiotherapy (CCRT) yielded better progression-free survival and overall survival (OS) with no significant increase in toxicity relative to neoadjuvant chemotherapy [7].

However, few studies have focused on the efficacy and safety of radical gastrectomy in patients following neoadjuvant CCRT, likely because surgical challenges after chemoradiotherapy increase due to the loss of normal tissue, the presence of plane-induced fibrotic reactions in tissue, and the effects of edematous changes. In this paper, we present our preliminary treatment results and compare the surgical outcomes and safety of neoadjuvant CCRT with those of chemotherapy alone in patients with LAGC (clinical stage cT3N2+, cT4aN+, or cT4b) in a single institution.

## 2. Materials and Methods

### 2.1. Patient Characteristics

**Figure 1:**
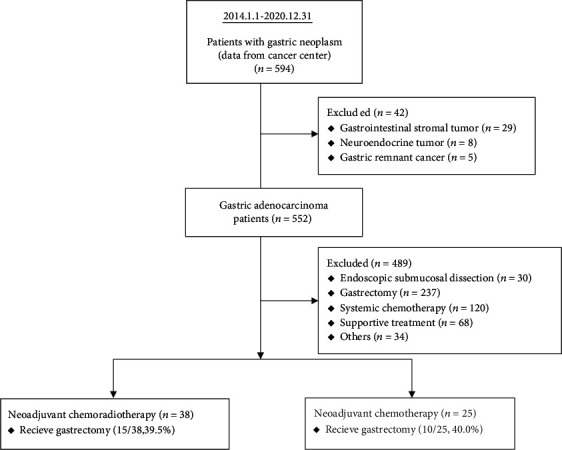
CONSORT flowchart illustrating the selection of 594 patients with gastric neoplasms, whose data were collected from the cancer center in our institution (January 1, 2014, to December 31, 2020).

We enrolled 594 patients with gastric cancer from a single institution who had received a relevant diagnosis between January 2014 and December 2020. Patients with a history of gastrointestinal stromal tumor (*n* = 29), neuroendocrine tumor (*n* = 8), gastric remnant cancer (*n* = 5), endoscopic submucosal dissection (*n* = 30), gastrectomy (*n* = 237), systemic chemotherapy (*n* = 120), or the best available supportive care (*n* = 68) were also excluded. Moreover, 34 patients in the “others” group were excluded due to loss to follow-up or treatment in other hospitals. Finally, 38 (38/63, 60.3%) patients consecutively diagnosed as having LAGC with neoadjuvant CCRT and 25 (25/63, 39.7%) patients diagnosed as having LAGC with neoadjuvant chemotherapy were enrolled; Figure 1 presents the flowchart of patient selection. A case series analysis was performed using data from a routinely updated and maintained electronic medical record database. Data on diagnosis, sex, clinical stage of the condition, and chemotherapy regimen were collected. All aspects of this study were approved by the Institutional Review Board of Kaohsiung Medical University Hospital (KMUHIRB-E(II)-20190122). After the inclusion and exclusion criteria were applied, this study retrospectively reviewed 49 patients with LAGC receiving neoadjuvant therapy, who gave their written informed consent. The responses of treatment were evaluated by a radiologist according to RECIST Version 1.1 [8], and tumor–node–metastasis (TNM) staging classification for carcinoma of the stomach was performed in accordance with the recommendations of the American Joint Committee on Cancer (AJCC Cancer Staging Manual 8th ed., 2017) [9].

### 2.2. Neoadjuvant Concurrent Chemoradiotherapy and Chemotherapy

The selection of neoadjuvant CCRT or chemotherapy depended on a shared decision-making framework implemented in our hospital. Thirty-three (33/38, 86.8%) patients receiving neoadjuvant CCRT and 19 (19/25, 76.0%) patients receiving neoadjuvant chemotherapy were administered a chemotherapy regimen with mFOLFOX4. On day 1, leucovorin (200 mg/m^2^) and oxaliplatin (85 mg/m^2^) were administered over 2 h. Subsequently, fluorouracil was continuously infused over 48 h at a dose of 2400 mg/m^2^; this happened once every 2 weeks. Dose reductions or interruptions were allowed if serious adverse events (≥grade 3 toxicity) occurred. The duration and dosage of radiotherapy were the same as those in our previous study [7].

### 2.3. Evaluation of Operative Morbidities

Patients were followed until July 2021 (median: 23.4 months, range: 10.1–60.5 months). Perioperative complications were evaluated using the Clavien–Dindo classification [10]. Readmission was defined as hospitalization within the postoperative 30 days.

### 2.4. Statistical Analysis

Data were analyzed using SPSS v20.0 (SPSS, Chicago, IL, USA). Bivariate analysis was first performed an intergroup comparison. Additionally, Fisher's exact test, an unpaired two-tailed *t-*test, and the McNemar test were used when appropriate. Statistical significance was set at *p* < 0.05.

## 3. Results

### 3.1. Patient Series, Tumor Characteristics, and Chemotherapy Regimen

Among the 63 patients with LAGC receiving neoadjuvant therapy included in our analysis (Table 1), 38 (38/63, 60.3%) patients were subsequently treated with neoadjuvant CCRT and 25 (25/63, 39.7%) patients were treated with neoadjuvant chemotherapy. The median ages of members of the CCRT and chemotherapy groups were 64.0 years (range: 34–83 years) and 71.0 years (range: 48–79 years), respectively. The status of patients receiving CCRT or chemotherapy was not related to tumor invasion (T stage, *p* = 0.277), lymph node metastasis (N stage, *p* = 0.741), or clinical stage of the disease (*p* = 0.277). Among the 63 patients, 38 (33/38, 86.8%) patients receiving neoadjuvant CCRT and 25 (19/25, 76.0%) patients receiving neoadjuvant chemotherapy were administered a chemotherapy regimen with mFOLFOX4.

### 3.2. Operative Details and Morbidities

After neoadjuvant therapy, 15 (15/38, 39.5%) patients in the CCRT group and 10 (10/25, 40.0%) patients in the chemotherapy group underwent radical gastrectomy (Table 2). Patients receiving neoadjuvant CCRT and patients receiving neoadjuvant chemotherapy did not significantly differ in operation time or intraoperative blood loss (both *p* > 0.05). Only one patient in the CCRT group received a blood transfusion during operation. The pathologic report revealed favorable tumor response and downstaging in the CCRT group after treatment: three patients exhibited a pathologic complete response (pCR) and four, seven, and one patients exhibited a condition at pathologic stages I, II, and III, respectively. By contrast, among the chemotherapy group patients, one patient exhibited pCR and one, four, and four patients exhibited a condition at pathologic stages I, II, and III, respectively.

For operative morbidities (Table 3), the CCRT group had a nonsignificantly higher rate of complications than the chemotherapy group (60.0% vs. 40.0%, *p* = 0.428), but most of the complications were mild. Most complications that appeared in the CCRT group were of Clavien–Dindo classification grade II. However, the mean postoperative length of stay did not significantly differ between the CCRT and chemotherapy groups (11.8 vs. 12.0, *p* = 0.206). No unplanned reoperation or death within 30 days after gastrectomy occurred in either group, and only one patient in the CCRT group was readmitted within the first 30 days after the operation (due to pneumonia).

## 4. Discussion

Gastric cancer is the sixth most common form of cancer in the world and the third most common cause of cancer death [1, 11]. In the past few decades, the survival rate of LAGC has remained poor even after curative surgery and adequate lymph node dissection. Neoadjuvant therapy can be used to make unresectable gastric cancer resectable (through downstaging) and increase the pCR rate of patients [4]. In Taiwan, more than 60% of patients with gastric cancer have advanced or metastatic gastric cancer at initial diagnosis, and curative surgery after neoadjuvant therapy might play a key role in treating such patients [12].

The MAGIC study revealed that in treating advanced gastric cancer, neoadjuvant chemotherapy with epirubicin, cisplatin, and fluorouracil (ECF) improves survival more than the sole use of surgery [5]. In a previous controlled, open-label, phase 2/3 trial, 716 patients with locally advanced resectable gastric and gastroesophageal junction cancer received perioperative fluorouracil plus leucovorin, oxaliplatin, and docetaxel (FLOT); they had a better OS compared with patients who received perioperative ECF and epirubicin, cisplatin, and capecitabine treatments [13]. In Western European countries, perioperative chemotherapy with FLOT has become the standard for treating locally advanced resectable gastric cancer. In a meta-analysis of randomized trials, including 15 randomized control trials and a total of 2001 patients with advanced gastric cancer and gastroesophageal junction cancer, neoadjuvant chemotherapy was found to reduce the mortality of gastric and gastroesophageal junction cancer and reduce the overall recurrence rate of gastroesophageal junction cancer [14].

Neoadjuvant CCRT is safe, effective, and feasible as a treatment for esophageal and rectal cancer, as indicated in its higher resection rates in numerous reports [15–19]. For patients with unresectable or borderline resectable gastric cancer who cannot undergo curative surgery, low resectability is the main cause of poor prognosis. Therefore, to improve oncologic outcomes, resection rates must be improved and neoadjuvant therapy must play a major role in conversion therapy. In our previous retrospective study on unresectable locally advanced or metastatic gastric cancer, neoadjuvant CCRT was associated with a higher response rate in the CCRT group (33.3%) than in the chemotherapy alone group (22.9%) [7]; furthermore, both groups exhibited similar levels of toxicity (all *p* > 0.05) [7]. In the present study, 15 (15/38, 39.5%) patients in the CCRT group received radical gastrectomy and 10 (10/25, 40.0%) patients in the chemotherapy group received radical gastrectomy after neoadjuvant therapy. Although the conversion rate did not significantly differ between the CCRT and chemotherapy groups, the pathologic findings indicated improved tumor response and downstaging in the CCRT group than in the chemotherapy group. Radiotherapy can be added to preoperative chemotherapy to achieve better local control and reduce locoregional recurrence [20].

Neoadjuvant chemotherapy has been demonstrated to be safe and to have similar outcomes as those of surgery alone. The Japan Clinical Oncology Group Study (JCOG0501) reported that neoadjuvant chemotherapy for type 4 and large type 3 gastric cancer followed by D2 gastrectomy can be safely performed, with no increases to morbidity or mortality [21]. Furthermore, laparoscopic distal gastrectomy is also safe, and it has a lower complication rate compared with open distal gastrectomy, for patients with LAGC following neoadjuvant chemotherapy [22].

However, the influence of surgery on combined radiotherapy and preoperative chemotherapy has gained more attention recently. In our study, operation time and intraoperative blood loss did not significantly differ between the CCRT and chemotherapy alone groups, and only one patient in the CCRT group received intraoperative blood transfusion due to severe adhesion and some blood loss during adhesiolysis. Although the complication rate was higher in the CCRT group than in the chemotherapy alone group (60.0% vs. 40.0%), the difference was not statistically significant (*p* = 0.428). Most complications found in the CCRT group were of Clavien–Dindo classification grade II, but both groups did not significantly differ with respect to mean postoperative length of stay (11.8 vs. 12.0, *p* = 0.206). No deaths or unplanned reoperations within 30 days after gastrectomy occurred in either group, and only one patient in the CCRT group was readmitted within 30 days of their operation due to pneumonia.

This study had the following limitations. First, only a few patients had preliminary results, which might have led to underestimated results. Second, our sample was small. Thus, future studies should use a larger sample and monitor patients over a relatively long period.

## 5. Conclusions

The effective treatment of patients with LAGC is challenging following preoperative neoadjuvant CCRT. Our present study revealed that patients with neoadjuvant CCRT had a favorable pathologic response without an increase in postoperative complications. However, further prospective randomized trials on patients with LAGC receiving neoadjuvant CCRT should be conducted to verify the findings of our retrospective study.

## Figures and Tables

**Table 1:** Patient characteristics at diagnosis and treatment (*n* = 63).

Characteristic	CCRT (*n* = 38)	Chemotherapy (*n* = 25)	*p*
Median age (years)	64.0	71.0	0.459
Gender (M : F)	27 : 11	15 : 10	0.419
Tumor invasion			0.277
cT3 or cT4a	23	19	
cT4b	15	6	
Lymph node metastasis			0.741
cN0 or cN1	6	5	
≧cN2	32	20	
cStage			0.277
III	23	19	
IVA	15	6	
Chemotherapy regimen			
FOLFOX4	33	19	
Cisplatin+5-FU	1	0	
XELOX	1	2	
Xeloda only	0	1	
Gemcitabine+S-1	1	0	
FLOT	2	3	

TNM staging classification for carcinoma of the stomach (American Joint Committee on Cancer, AJCC 8^th^ ed., 2017). FOLFOX: folinic acid+fluorouracil+oxaliplatin; 5-FU: 5-fluorouracil; XELOX: capecitabine+oxaliplatin; Xeloda: capecitabine; S-1: tegafur/gimeracil/oteracil; FLOT: fluorouracil+folinic acid+oxaliplatin+docetaxel.

**Table 2:** Operative detail.

	CCRT (*n* = 15)	Chemotherapy (*n* = 10)	*p*
Median age (years)	65.0	71.0	0.385
Operation time (min)			
Mean	303.9	326.0	0.241
Blood loss (mL)			
Mean	216.3	182.2	0.324
Surgery			
Proximal gastrectomy	1	0	
Distal gastrectomy	12	7	
Total gastrectomy	2	3	
Blood transfusion (yes)	1	0	
ypTNM stage			
0	3	1	
I	4	1	
II	7	4	
III	1	4	
IV	0	0	

TNM staging classification for carcinoma of the stomach (American Joint Committee on Cancer, AJCC 8^th^ ed., 2017).

**Table 3:** Operative morbidities.

	CCRT (*n* = 15)	Chemotherapy (*n* = 10)	*p*
Postoperative complications	9 (60.0%)	4 (40.0%)	0.428
Clavien–Dindo classification			
Grade I	0	0	
Grade II	8 (53.3%)	3 (30.0%)	0.414
Pneumonia	2	1	
Abscess	3	1	
Urinary tract infection	2	0	
Gastroparesis	1	1	
Digestive tract hemorrhage	2	0	
Wound infection	1	0	
Grade IIIa	2 (13.3%)	0	0.500
Pleural effusion	2	0	
Grade IIIb	0	0	
Grade IV	0	1 (16.7%)	0.400
Acute myocardial infarction	0	1	
Intraperitoneal hemorrhage	0	1	
Postoperative length of stay, mean (day)	11.8	12.0	0.206
Readmission within postoperative 30 days (yes)	1	0	0.400

## Data Availability

The datasets used and/or analyzed during the current study are available from the corresponding author on reasonable request.
